# Functional Connectivity Within the Executive Control Network Mediates the Effects of Long-Term Tai Chi Exercise on Elders’ Emotion Regulation

**DOI:** 10.3389/fnagi.2018.00315

**Published:** 2018-10-23

**Authors:** Zhiyuan Liu, Yuyan Wu, Lin Li, Xiuyan Guo

**Affiliations:** ^1^School of Psychology, Shaanxi Normal University, Xi’an, China; ^2^Shanghai Key Laboratory of Magnetic Resonance, School of Physics and Materials Science, East China Normal University, Shanghai, China; ^3^School of Psychology and Cognitive Science, East China Normal University, Shanghai, China; ^4^National Demonstration Center for Experimental Psychology Education, East China Normal University, Shanghai, China; ^5^Shanghai Key Laboratory of Brain Functional Genomics, Key Laboratory of Brain Functional Genomics, Ministry of Education, School of Psychology and Cognitive Science, East China Normal University, Shanghai, China

**Keywords:** tai chi, meditation, emotion regulation, executive control network, resting-state functional connectivity, functional magnetic resonance imaging

## Abstract

Previous research has identified the effects of tai chi exercise on elders’ executive control or on their emotion regulation. However, few works have attempted to reveal the relationships between tai chi, executive control, and emotion regulation in the same study. The current resting-state study investigated whether the impact of tai chi on elders’ emotion regulation was mediated by the resting-state functional connectivity within the executive control network. A total of 26 elders with long-term tai chi experience and 26 demographically matched healthy elders were recruited. After the resting-state scan, both groups were required to complete a series of questionnaires, including the Five Facets Mindfulness Questionnaire (FFMQ), and a sequential decision task, which offered an index of the subjects’ emotion-regulation ability by calculating how their emotional response could be affected by the objective outcomes of their decisions. Compared to the control group, the tai chi group showed higher levels of non-judgment of inner experiences (a component of the FFMQ), stronger emotion-regulation ability, and a weaker resting-state functional connectivity between the dorsolateral prefrontal cortex (DLPFC) and the middle frontal gyrus (MFG). Moreover, the functional connectivity between the DLPFC and the MFG in the tai chi group fully mediated the impact of non-judgment of inner experience on their emotion-regulation ability. These findings highlighted that the modulation of non-judgment of inner experience on long-term tai chi practitioners’ emotion regulation was achieved through decreased functional connectivity within the executive control network.

## Introduction

Emotional suffering in older adults decreases their quality of life and increases their risk for mental disease, such as depression and anxiety (Berkman et al., 1986; Rozzini et al., 2001). A growing body of research has demonstrated that emotional suffering in older adults is associated with age-related cognitive impairment (Lesser et al., 1996; Butters et al., 2004), which is reflected by changes in a series of indicators such as gray matter volume, brain activity during tasks, and brain functional connectivity in the resting state. The pathology of brain networks in cognitive impairment in older adults has been investigated by applying resting-state functional connectivity (Liu et al., 2015; Liu F. et al., 2017; Lin et al., 2018). Most of these studies revealed that a key brain network in cognitive impairment was the executive control network, which influences many domains of life, such as academic success and healthy eating (Robinson et al., 2010; Hofmann et al., 2012). The prevention of declining executive control appears to be important and should become one of the best long-term strategies to achieve successful aging.

The organization and function of the human brain throughout life is plastic (Kurth et al., 2015). Evidence regarding the plasticity of executive control function can be demonstrated by mind-body training (Tang et al., 2015). The most well-researched mind-body training for executive control function is meditation (Teper and Inzlicht, 2013), which is thought to be able to promote executive control function through improving present-moment awareness and non-judgmental acceptance, thus achieving increased sensitivity to affective cues that help signal the need for control (Cardaciotto et al., 2008; Teper et al., 2013). Thus, it is not surprising that there is a wealth of evidence to support the association between meditation training and improved executive control function on both behavioral and neural levels (Hölzel et al., 2011; Zanesco et al., 2013). For example, Wenksormaz (2005) found that engaging in meditation training improved executive control function. Tao et al. (2017) recently revealed that meditation training enhanced executive control function by decreasing the resting-state functional connectivity between the key regions such as the dorsolateral prefrontal cortex (DLPFC) and the frontal regions in the central executive control network. Researchers found that compared to the meditation training group, older adults in the control group might compensate for disruption of the executive control network by recruiting additional frontal resources to overcome executive control deficits (Gutchess et al., 2007; Tao et al., 2017). Therefore, meditation training can reduce compensation for executive control function, which was reflected by weaker functional connectivity within the executive control network among meditators. Moreover, meditation was often described as non-judgmental acceptance or regulation of the present emotional experiences (Teper et al., 2013). Improvements in emotion regulation associated with meditation have been investigated through self-reporting and physiological and neuroimaging methods (Fox et al., 2012; Tang and Posner, 2012). For instance, Ortner et al. (2007) found that meditators represented reduced emotional responsiveness to unpleasant situations, suggesting an enhancement in emotion regulation to avoid the potentially harmful effects of negative emotions. Taken together, prior studies have shown the remarkable influence of meditation on both executive control function and emotion regulation. However, these previous studies failed to provide direct evidence for the relationship among meditation, executive control, and emotion regulation. The current report aimed to explore this issue.

Meditation encompasses a family of complex practices that include mindfulness meditation, yoga, and tai chi (Tang et al., 2015). Of these, tai chi, a multimodal mind-body exercise that incorporates physical, cognitive, and meditative components in the same activity is growing in popularity, especially among older adults (Wayne et al., 2014). Previous studies have suggested that tai chi, as a physical exercise, was an effective method not only to improve health fitness, such as neuromuscular functions and cardiorespiratory system and balance control (Ray et al., 2005; Wang et al., 2010; Ghaffari and Kluger, 2014), but also benefit emotion regulation and psychological well-being in elders (Wang et al., 2010). The current resting-state functional magnetic resonance imaging (fMRI) study (containing a tai chi group and a control group) aimed to replicate previous findings that meditative components in tai chi were related to enhanced executive control and stronger emotion regulation. More importantly, taking the functional connectivity of the executive control network in the resting state as an indicator of executive control, we tried to assess whether tai chi achieves inducing a stronger emotion-regulation ability via enhancing the function of the executive control network.

The main focus of the current study was to examine whether the impact of the meditative component of tai chi on emotion regulation was mediated by the functional connectivity between the DLPFC (a core region of the executive control network) (Sheline et al., 2010) and the frontal regions. Behaviorally, we predicted that the tai chi group would have a higher meditation score and stronger emotion regulation than the control group. At the neural level, in line with previous findings, we predicted that the tai chi group would show weaker functional connectivity between the DLPFC and the frontal regions, such as the middle frontal gyrus (MFG) in the resting state. Finally, we predicted that the impact of meditation on emotion regulation in the tai chi group was mediated by functional connectivity between the DLPFC and the MFG.

## Materials and Methods

### Subjects

Totally 26 tai chi practitioners and 26 control subjects were recruited from the community. The subjects in the tai chi group had engaged in tai chi for an average of 10.44 ± 5.48 years. The control participants were active in other types of physical exercise without a meditation component, such as jogging and square dancing. The subjects’ demographic characteristics are provided in Table 1. All of the subjects (1) did not have any neurological diseases, history of stroke, or severe cerebrovascular diseases, (2) had normal or corrected-to-normal vision, (3) had the ability to provide written informed consent, and (4) were right-handed. All of the subjects provided written informed consent before the study began. One control subject was excluded due to severe head motion (>2 mm or 2°). The remaining 26 tai chi subjects and 25 control subjects were included in the data analyses. This study was approved by the Ethics Committee on Human Experiments of East China Normal University. The protocol was approved by the Ethics Committee on Human Experiments of East China Normal University. All subjects gave written informed consent in accordance with the Declaration of Helsinki.

**Table 1:** Demographics of the tai chi and control groups.

Characteristics	Age (years)	Gender (male/female)	Education (years)	Tai chi (years)	Exercise time per day (min)
Tai chi group	65.19 ± 2.30	8/18	10.46 ± 1.79	10.44 ± 5.48	66.76 ± 20.51 (Tai chi)
Control group	63.92 ± 2.87	9/16	11.04 ± 2.57	0	61.54 ± 25.62 (Other exercise)
*t*	1.751	0.157	-0.934	NA	0.804
*p*	0.086	0.692	0.355	NA	0.425


### Procedures

#### Questionnaires

Before scanning, the subjects were required to complete the Chinese version of the Beck Depression Inventory (BDI) (Beck et al., 1987; Kurylo and Stevenson, 1992), the NEO Five-Factor Inventory (NEO-FFI) (Kurylo and Stevenson, 1992), the Five Facets Mindfulness Questionnaire (FFMQ) (Baer et al., 2006), and the Mindful Attention Awareness Scale (MAAS) (Brown and Ryan, 2003).

##### Beck Depression Inventory

The BDI was used to assess the depression level of participants. The BDI included 21 items, which was used to measure the symptoms associated with depression. The split-half coefficient of the Chinese version of the BDI was 0.879 and Cronbach’s alpha was 0.890. The BDI and its individual items were shown to have good construct and concurrent validities in China (Zhang, 1990).

##### NEO Five-Factor Inventory

The personality of participants was measured by using the NEO-FFI, a questionnaire addressing five core personality traits: neuroticism, extraversion, openness, conscientiousness, and agreeableness. Each dimension consisted of 12 statements. Participants were asked to rate the degree to which they agree with these statements. Each statement was rated on a 5-point scale (1 = completely agree, 5 = completely disagree), yielding a scale score ranging from 12 to 60.

##### Five Facets Mindfulness Questionnaire

The FFMQ was used to assess the meditation level of the participants. The FFMQ consisted of 39 items that were rated on a 5-point Likert-type scale (1 = never or very rarely true, 5 = very often or always true). This scale measures five distinct facets of mindfulness: (1) observing (defined in terms of noticing or attending to internal and external experiences, e.g., I notice the smells and aromas of things), (2) describing (defined in terms of labeling internal experiences with words, e.g., I am good at finding words to describe my feelings), (3) acting with awareness (defined in terms of attending to one’s activities of the moment, e.g., reverse-scoring item: I am easily distracted), (4) non-judgmental of inner experience (defined in terms of taking a non-evaluative stance toward thoughts and feelings, e.g., reverse scoring item: I disapprove of myself when I have irrational ideas), and (5) non-reactivity to inner experience (defined in terms of allowing thoughts and feelings to come and go, without getting caught up in or carried away by them, e.g., I watch my feelings without getting lost in them). Baer et al. (2006) concluded that the FFMQ had an adequate-to-good internal consistency with the following Cronbach coefficients: observing = 0.83, describing = 0.91, acting with awareness = 0.87, non-judgmental of inner experience = 0.87, and non-reactivity to inner experience = 0.75. The Chinese version of the FFMQ had acceptable psychometric properties and was a valid instrument for the assessment of mindfulness (Deng et al., 2011).

##### Mindful Attention Awareness Scale

The MAAS was a 15-item instrument measuring the general tendency to be attentive to and aware of present-moment experience in daily life. It had a single factor structure and yielded a single total score. Using a 6-point Likert-type scale (almost always to almost never), respondents rate how often they have experiences of acting on automatic pilot, being preoccupied, and not paying attention to the present moment. Brown and Ryan (2003) reported an internal consistency (coefficient alpha) of 0.82 and expected convergent and discriminant validity correlations. The MAAS and its individual items were shown to have good construct and concurrent validities in China (Deng et al., 2012).

### Tasks

The results of the resting-state scans used in the current study were collected immediately after the anatomical scan. The duration of the resting-state scan was 8 min and 6 s. The subjects were asked to stay awake and remain motionless with their eyes closed and ears plugged during the resting-sate scan.

After the resting-state scan, the subjects were instructed to undertake a sequential decision task (see the experimental processes in Brassen et al., 2012). The sequential decision task was proven to be solid by previous studies (Brassen et al., 2012; Liu et al., 2016; Liu F. et al., 2017; Li et al., 2018). By using this task, we acquired information on the subjects’ emotion changes to different outcomes. The subjects were informed that they would obtain tokens (gold coins) from the task and that payment for their participation was determined by the total number of tokens they obtained (1 token for 1 Chinese yuan).

All of the subjects participated in 80 trials of the sequential decision task. For each trial, eight boxes were presented, seven containing gold coins and one containing a devil. The position of the devil was set randomly at each trial. The subjects were instructed to open the boxes from left to right and stop when they wanted to collect the coins acquired thus far. They had to decide whether to open the next box or collect their coins within 2 s by pressing a button. Exposing the randomly distributed devil ended the trial, and all of the tokens from the trial were lost. If the subjects stopped and collected their gains, the position of the devil was revealed, thus informing the subjects about both the number of gold coins they gained and the number they had missed. The outcome of each trial was one of the following two conditions: (1) a gain condition, in which the subjects did not unpack the devil and gained gold coins in that trial and (2) a loss condition, in which the subjects unpacked the devil and lost the gold coins collected in that trial. A jittered interval was presented either after the subjects decided to stop or after they unpacked the devil. Next, the outcome was presented for 3 s. This was highlighted on a screen by a cyan square (in the case of stopping and collecting the gains) or by a red square (in the case of unpacking the devil and losing the gains in that trial). Finally, the emotional rating stage was presented. At this stage, the subjects were asked to rate how they felt about their choices on a 9-point scale from extreme regret (defined as -4) to extreme relief (defined as 4) in 3 s.

### fMRI Data Acquisition

The fMRI data were acquired using a 3.0-T Siemens Trio system scanner (East China Normal University, Shanghai, China). Prior to the resting-state stage, a high-resolution structural image was acquired using a T1-weighted, multiplanar reconstruction (MPR) sequence [repetition time (TR) = 1900 ms, echo time (TE) = 3.42 ms, 192 slices, slice thickness = 1 mm, field of view (FOV) = 256 mm, matrix size = 256 × 256]. Resting-state fMRI data were acquired using a gradient-echo echo-planar imaging (EPI) sequence (TR = 2000 ms, TE = 30 ms, FOV = 220 mm, matrix size = 64 × 64, 35 slices, slice thickness = 4 mm).

### Data Analyses

#### Demographic Characteristics and Scale Data Analyses

To investigate the different demographic characteristics and scale scores between the two groups, independent samples *t*-tests and Chi-squared tests were conducted using SPSS 18.0 software. A threshold of *p* < 0.05 (two-tailed) was applied.

#### Behavioral Data Analyses

In the gain condition, we calculated a combined index, called the real gain percentage (RGP), which was defined as the ratio of the collected gains and the largest possible gains (that is, the total number of boxes before the devil) in a given trial (Liu et al., 2016; Liu Z. et al., 2017; Li et al., 2018). The RGP value can be considered an indication of how good the outcome is in a particular trial. Regression analyses were performed for each subject to investigate the differences in the subjects’ sensitivity to the objective outcomes between the two groups. The RGP value was defined as a predictor and emotional ratings as independent variables (see the following equation).

Emotional rating = K × RGP value + b

Each subject’s regression coefficient (K) and intercept (b) were calculated. K was considered an index of their sensitivity to the objective outcomes in the gain condition. Independent samples *t*-tests in SPSS 18.0 software were performed to investigate the differences between the two groups in emotional stability. A threshold of *p* < 0.05 (two-tailed) was applied.

In the loss condition, we also performed regression analyses for each subject in which the number of lost coins was defined as a predictor and the emotional ratings as independent variables. Independent samples *t*-tests in SPSS 18.0 software were performed to compare the differences in regression coefficients of the two groups. A threshold of *p* < 0.05 (two-tailed) was applied.

#### fMRI Data Preprocessing

Data preprocessing was performed using the Data Processing Assistant for Resting-State fMRI Advanced (DPARSFA^1^) in MATLAB. The DPARSFA software was based on Statistical Parametric Mapping (SPM8^2^) and the Resting-State fMRI Data Analysis Toolkit (REST^3^). The first 10 volumes were not analyzed to allow for the signal equilibration of each subject. The remaining 230 time points from each subject were corrected for the delay in slice acquisition. Afterward, the images were realigned and head motions corrected and coregistered to the respective T1-weighted structural images of each subject. The coregistered structural images were then segmented into gray matter (GM), white matter (WM), and cerebrospinal fluid (CSF) using a unified segmentation algorithm. Next, the 6 rigid body motion parameters, WM, and CSF signals were regressed out. The functional images were spatially normalized to the Montreal Neurological Institute (MNI) space (resampled to 2 mm × 2 mm × 2 mm) using the normalization parameters estimated during unified segmentation and spatially smoothed with a Gaussian kernel of 6-mm full-width half-maximum (FWHM) and linearly detrended. Finally, the data were bandpass filtered from 0.01 to 0.08 Hz.

#### fMRI Data Analyses

Given the importance of the DLPFC to executive function, we chose the DLPFC (seed region, MNI 36 27 29) as the region of interest based on previous research that demonstrated that the DLPFC was a vital region in the CCN network (Fales et al., 2008; Sheline et al., 2010; Hwang et al., 2015). For each subject, seed-based FC was computed as the Pearson correlation coefficients between the seed region (a 6-mm sphere around the coordinates of the DLPFC) and other voxels of the whole brain. The correlation coefficients were then z-transformed for standard purposes (the fisher r to z), and seed-based FC maps were generated. Group differences were compared using voxel-wise independent sample *t*-tests. Moreover, to explore the association between the resting-state functional connectivity and behavioral variables (meditation score and *K*-value), we also performed regression analyses. For all of the fMRI analyses, age, gender, and years of education were included in the analysis as covariates of non-interest. Finally, a cluster-level threshold of *p* < 0.05 (familywise error, FWE) and a voxel-level threshold of *p* < 0.005 (uncorrected) were used to define activations.

## Results

### Demographic Data and Scale Data

Two groups showed no significant differences in demographics, such as age [*t*_(49)_ = 1.751, *p* = 0.086], gender [*t*_(49)_ = 0.157, *p* = 0.692], education [*t*_(49)_ = -0.934, *p* = 0.355], and physical exercise time per day [*t*_(49)_ = 0.804, *p* = 0.425] (Table 1). The tai chi group scored higher on non-judgment of inner experience [*t*_(49)_ = 2.336, *p* < 0.05], non-reactivity [*t*_(49)_ = 3.097, *p* < 0.01], total FFMQ scores [*t*_(49)_ = 2.277, *p* < 0.05], and MAAS scores [*t*_(49)_ = 2.447, *p* < 0.05] relative to the control group. No other significant difference in scales was found between the two groups (Table 2).

**Table 2:** Scale data of the tai chi and control groups.

Psychological measures		Tai chi group	Control group	*t*	*p*
**BDI**	
	Depressive	4.69 ± 5.36	5.36 ± 4.86	–0.465	0.644
**NEO-FFI**	
	Neuroticism	27.31 ± 7.62	30.92 ± 6.19	–1.854	0.070
	Extraversion	42.1 ± 6.74	40.76 ± 4.90	0.865	0.391
	Openness	39.77 ± 5.14	39.64 ± 4.64	0.634	0.529
	Agreeableness	45.11 ± 6.57	44.16 ± 5.69	0.554	0.582
	Conscientiousness	48.27 ± 5.65	48.04 ± 5.04	0.153	0.879
**FFMQ**	
	Observing	3.50 ± 0.79	3.21 ± 0.62	1.427	0.160
	Describing	3.55 ± 0.66	3.48 ± 0.50	0.415	0.680
	Act with awareness	3.68 ± 0.95	3.74 ± 0.70	–0.267	0.791
	Non-judging	2.86 ± 0.49	2.55 ± 0.46	2.336	0.024^∗^
	Non-reactivity	3.58 ± 0.46	3.11 ± 0.60	3.097	0.003^∗∗^
	Total FFMQ score	17.16 ± 1.83	16.10 ± 1.48	2.277	0.027^∗^
**MAAS**	
	Total MAAS score	72.54 ± 11.72	63.64 ± 14.18	2.447	0.018^∗^


*BDI, Beck Depression Inventory; NEO-FFI, NEO Five-Factor Inventory; FFMQ, Five Facets Mindfulness Questionnaire; MAAS, Mindful Attention Awareness Scale. ^∗^p < 0.05, ^∗∗^p < 0.01.*

### Behavioral Results

**FIGURE 1:**
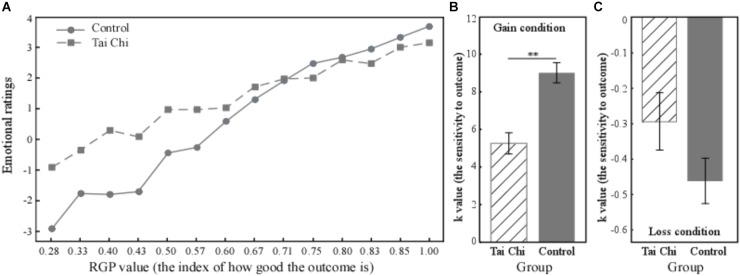
The relationship between the subjects’ emotional ratings and RGP in the tai chi group **(A)**. The independent samples *t*-test revealed that the regression coefficient (K) in the tai chi group was significantly smaller than in the control group [*t*_(49)_ = 4.82, *p* < 0.01] **(B)**. The regression coefficient (K) showed no significant difference between two groups in Loss condition [*t*(49) = 1.757, *p* = 0.085] **(C)**. ^∗∗^*p* < 0.01.

**FIGURE 2:**
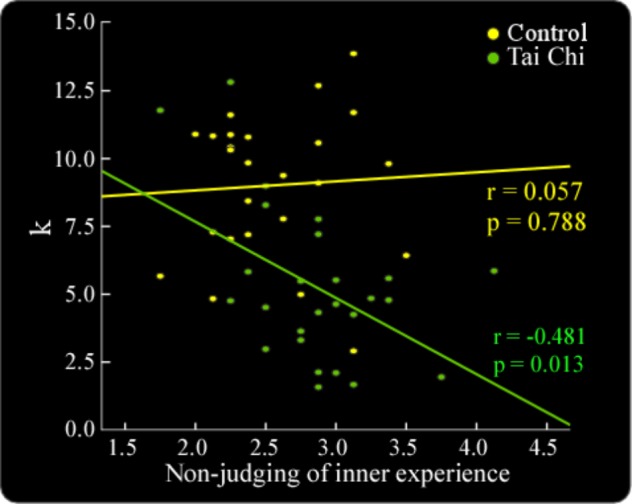
The correlation analyses showed that non-judgment of inner experience was negatively correlated with the *K*-value in the tai chi group (*r* = –0.481, *p* < 0.05). No such correlation was found in the control group (*r* = 0.057, *p* > 0.05).

In the gain condition, the relationships between the subjects’ emotional ratings and RGP in both the tai chi and control groups were described (Figure 1A). Moreover, independent samples *t*-tests showed that the *K*-value in the tai chi group was significantly smaller than in the control group [*t*_(49)_ = 4.82, *p* < 0.01] (Figure 1B). This result indicated that the tai chi group showed less sensitivity to the objective outcomes than the control group. In addition, the result showed non-judgment of inner experience was negatively correlated with the *K*-value across all subjects (*r* = -0.365, *p* < 0.05). Further analysis found the significant correlation between non-judgment of inner experience and *K*-value in the tai chi group (*r* = -0.481, *p* < 0.05). We did not find such a correlation in the control group (*r* = 0.057, *p* > 0.05) (Figure 2).

In the loss condition, the regression coefficient in the tai chi group had a tendency to be smaller relative to the control group, although this did not reach statistical significance [*t*_(49)_ = 1.757, *p* = 0.085] (Figure 1C).

### fMRI Results

#### Independent Samples *t*-Tests

**FIGURE 3:**
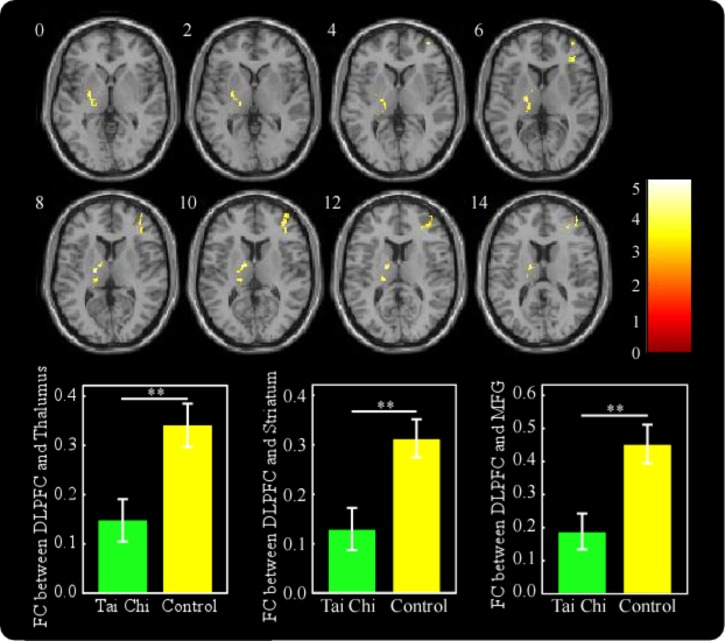
The tai chi group showed significantly decreased resting-state functional connectivity between the DLPFC and the left thalamus (MNI –20 –14 6), left ventral striatum (MNI –26 –8 2), and right MFG (MNI 3848 10) compared to the control group. ^∗∗^*p* < 0.01

Seed-based functional connectivity was computed as the Pearson correlation coefficients between the seed region (DLPFC) and other voxels of the whole brain. The results revealed that the subjects in the tai chi group showed significantly decreased resting-state functional connectivity between the DLPFC (seed region) and the left thalamus (MNI -20 -14 6), left ventral striatum (MNI -26 -8 2), and right MFG (MNI 3848 10) compared to the subjects in the control group (Figure 3 and Table 3). The subjects in the tai chi group did not show significantly stronger functional connectivity than those in the control group when the seed region was the DLPFC.

**Table 3:** The difference in resting-state functional connectivity of the tai chi and control groups.

		Peak activation		
Region		*X*	*Y*	*Z*	*t-*Value	Voxels
**Seed region: DLPFC**						
***Tai chi < control***						
L	Thalamus	–20	–14	6	4.16	165
L	Pallidum	–26	–8	2	3.93	
R	MFG	38	48	10	3.87	123
***Tai chi > control***						
	No region					


*Coordinates (mm) are in MNI space. L, left hemisphere; R, right hemisphere. All of the clusters survived FWE correction (p < 0.05) for multiple comparisons at the cluster level, with a voxel-level threshold corresponding to p < 0.005, uncorrected.*

#### Correlation Analyses

**FIGURE 4:**
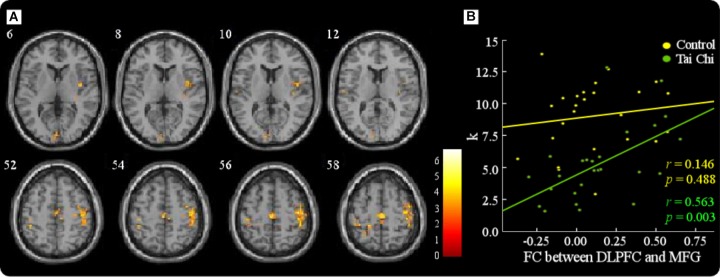
In the tai chi group, non-judgment of inner experience had a negative association with functional connectivity between the DLPFC and the right MFG (MNI 44 –4 60) **(A)**. The results showed that the functional connectivity strength between the DLPFC and the MFG was negatively correlated with non-judgment of inner experience in the tai chi group (*r* = –0.505, *p* < 0.01). No correlation was found in the control group (*r* = 0.07, *p* > 0.05) **(B)**.

**FIGURE 5:**
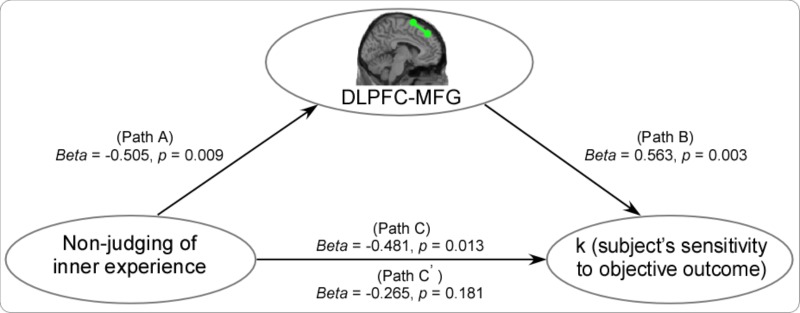
The *K*-value and the functional connectivity between the DLPFC and the right MFG (MNI 38 –6 54) showed a positive correlation in the tai chi group **(A)**. The results showed that the functional connectivity strength between the DLPFC and the MFG was positively correlated with the *K*-value in the tai chi group (*r* = 0.563, *p* < 0.01). No correlation was found in the control group (*r* = 0.146, *p* > 0.05) **(B)**.

In the tai chi group, non-judgment of inner experience was negatively associated with functional connectivity between the DLPFC and the right MFG (MNI 44 -4 60) (Figure 4A and Table 4). The functional connectivity strength between the DLPFC and the MFG (MNI 44 -4 60) was then calculated. In order to express the results more clearly, Figure 4B reveals that the functional connectivity strength between the DLPFC and the MFG was negatively correlated with non-judgment of inner experience (*r* = -0.505, *p* < 0.01). Moreover, the *K*-value and the resting-state functional connectivity between the DLPFC and the right MFG (MNI 38 -6 54) showed a positive correlation in the tai chi group (Figure 5A and Table 5). Figure 5B describes the relationship between the *K*-value and the DLPFC resting-state functional connectivity (*r* = 0.563, *p* < 0.01). No aforementioned correlation was found in the control group. In addition, both the functional connectivity strength between the DLPFC and the thalamus and the functional connectivity strength between the DLPFC and the ventral striatum did not show significant correlation with non-judgment of inner experience or the *K*-value.

**Table 4:** The correlation between non-judgment of inner experience and resting-state functional connectivity in the tai chi group (seed region: DLPFC).

		Peak activation		
Region		*X*	*Y*	*Z*	*t-*Value	Voxels
***Negatively correlated with non-judging of inner experience***						
L	Precentral gyrus	–38	–14	58	5.68	245
L	Superior frontal gyrus	–22	–8	74	3.48	
R	Precentral gyrus	64	–12	36	4.63	193
R	Rolandic operculum	58	–8	10	4.73	136
R	Precentral gyrus	42	–16	56	4.3	89
R	MFG	44	–4	60	3.54	
***Positively correlated with non-judging of inner experience***						
	No region					


*Coordinates (mm) are in MNI space. L, left hemisphere; R, right hemisphere. All of the clusters survived FWE correction (p < 0.05) for multiple comparisons at the cluster level, with a voxel-level threshold corresponding to p < 0.005, uncorrected.*

**Table 5:** The correlation between the *K*-value and resting-state functional connectivity in the tai chi group (seed region: DLPFC).

		Peak activation		
Region		*X*	*Y*	*Z*	*t-*Value	Voxels
***Positively correlated with k***						
R	Supramarginal gyrus	66	–20	26	6.81	1309
R	Insula lobe	40	–6	0	4.33	
R	MFG	38	–6	54	3.82	
L	Precentral gyrus	–62	–18	30	5.22	401
L	Inferior parietal lobule	–46	–24	38	5.01	
R	Paracentral lobule	10	–32	64	4.85	161
L	Postcentral gyrus	–36	–36	58	5.3	133
***Negatively correlated with K***						
	No region					


*Coordinates (mm) are in MNI space. L, left hemisphere; R, right hemisphere. All of the clusters survived FWE correction (p < 0.05) for multiple comparisons at the cluster level, with a voxel-level threshold corresponding to p < 0.005, uncorrected.*

#### Mediation Analyses

**FIGURE 6:**
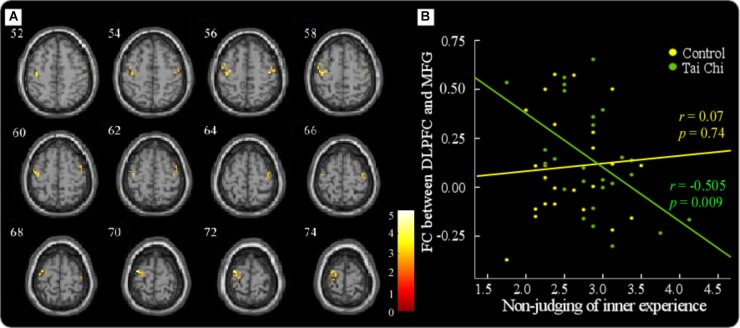
The mediation analysis showed that there was no significant effect of non-judgment of inner experience on the subjects’ sensitivity to outcomes (K) after including the functional connectivity between the DLPFC and the MFG (Path A: *beta* = –0.505, *p* < 0.01; Path B: *beta* = 0.563, *p* < 0.01; Path C: *beta* = –0.481, *p* < .05; Path c’: *beta* = –0.265, *p* > 0.05).

To test whether the effect of non-judgment of inner experience on the subjects’ sensitivity to outcomes (K) was mediated by the functional connectivity between the DLPFC and the MFG, we conducted a mediation analysis. The mediation analysis showed that there was no significant effect of non-judgment of inner experience on the subjects’ sensitivity to outcomes after including the functional connectivity between the DLPFC and the MFG (path A: *beta* = -0.505, *p* < 0.01; path B: *beta* = 0.563, *p* < 0.01; path C: *beta* = -0.481, *p* < .05; path c’: *beta* = -0.265, *p* > 0.05) (Figure 6). Thus, the functional connectivity between the DLPFC and the MFG fully mediated the impact of non-judgment of inner experience on the subjects’ sensitivity to outcomes.

## Discussion

In the current cross-sectional study, we combined resting-state fMRI and a sequential decision task to investigate whether the impact of the meditative component of tai chi on emotion regulation was mediated by resting-state functional connectivity within the executive control network. Behaviorally, the tai chi group showed a higher propensity to adopt a non-judgmental stance toward their inner experience and less sensitivity to outcomes (the reflection of stronger emotion-regulation ability) than the control group. Furthermore, we found that the non-judgment score of inner experience was negatively correlated with the subjects’ sensitivity to outcomes in the tai chi group. At the neural level, the tai chi group showed decreased functional connectivity between the DLPFC and the MFG compared to the control group. In addition, the functional connectivity between the DLPFC and the MFG was negatively correlated with non-judgment of inner experience and positively correlated with the subjects’ sensitivity to outcome only in the tai chi group. Interestingly, the functional connectivity between the DLPFC and the MFG fully mediated the impact of non-judgment of inner experience on the tai chi group’s sensitivity to outcome.

In line with our hypothesis, the results showed that long-term tai chi exercise was associated with enhanced non-judgment of inner experience and reduced sensitivity to outcomes. In the tai chi group, a strong stance to not judge inner experience might have mitigated the abstract thoughts evaluating the characteristics of outside stimuli, thereby reducing the intensity of emotional sensitivity to outcomes, that is, improving emotional stability. Thus, tai chi training might improve emotional stability in older adults as a reflection of stronger emotion regulation. For those in the tai chi group, non-judgment of inner experience was positively correlated with their emotional stability, supporting the notion that increased emotional stability may be an outcome of long-term meditation training (Tang et al., 2015, 2016). Previous work has shown that meditation was an effective way to improve individuals’ core psychological and cognitive abilities, including emotion regulation (Lutz et al., 2008; Moyer et al., 2011). Literature reported that the connection between meditation and improved emotion regulation was certainly intuitive regarding the emphasis on the non-judgmental acceptance of thoughts and emotions at the core of meditation training (KabatZinn, 2014). Long-term tai chi practitioners might learn to intentionally observe and accept affective states and were able to reduce habitual tendencies to ruminate about their feelings (Brown et al., 2013) as well as strengthen adaptive processing of emotional information (Farb et al., 2013). Taken together, our results suggested that long-term tai chi practice is an effective way to improve emotion regulation in older adults, where enhanced non-judgment of inner experience through tai chi training might play an important role.

Mind-body exercises such as tai chi and yoga can significantly enhance cognitive function by modulating the brain functioning and structures associated with cognitive processes (Wei et al., 2015; Afonso et al., 2017). In recent years, the popularity of resting-state functional connectivity has further endorsed this method of investigating the brain as a network (Li et al., 2014; Liu et al., 2015; Tao et al., 2016). The current study found that the tai chi group showed decreased DLPFC-MFG functional connectivity compared to the control group. This result was consistent with recent findings that tai chi practice significantly decreased resting-state functional connectivity between the DLPFC and the frontal regions (Tao et al., 2017). A large body of evidence indicated that the DLPFC and the MFG were key regions of the executive control network playing important roles in top-down cognitive control processes such as intellectual performance (Hopfinger et al., 2000; MacDonald et al., 2000), impression management (Vohs et al., 2005), and emotion regulation (Compton et al., 2008). Neuroimaging studies on the executive control network and aging suggested that high-performing older adults may compensate for disruption of the cognitive control network by recruiting additional frontal resources to overcome cognitive control deficits (Gutchess et al., 2007). For example, a study that investigated the effects of a 14-day longevity lifestyle program found that improved brain metabolism was associated with a decrease in the connectivity between the DLPFC and the frontal regions, which was interpreted as a marker of greater cognitive efficiency of this brain region (Small et al., 2006). Moreover, Tang et al. (2015) suggested that novice meditators need to overcome habitual ways of internally reacting to their own emotions and might therefore show an increased recruitment of the prefrontal regions, while experienced meditators (that is, those with long-term tai chi experience) might have automated an accepting stance toward their experience and therefore show weaker prefrontal activation. Hence, the benefits associated with increased activity within the executive control network might be related to compensatory mechanisms rather than indicating a healthy state. In the current study, decreased functional connectivity between the DLPFC and the MFG in older adults with long-term tai chi experience might be a desirable outcome, which suggested the improvement and high efficiency of executive control.

Healthy elders in both the tai chi and control groups were balanced in age, gender, educational years, and personality traits such as depression and impulsivity. Therefore, it was unlikely that the resting-state functional connectivity difference observed in the executive control network between the groups was due to the demographic characteristics. Importantly, the DLPFC-MFG functional connectivity was negatively correlated with non-judgment of inner experience, a key component of meditation, among elders with long-term tai chi experience, indicating that a stronger meditation level was associated with weaker functional connectivity within the executive control network. In fact, many studies have confirmed the positive benefits of meditation such as improving cognitive flexibility (Moore and Malinowski, 2009; Kozasa et al., 2012). For example, Amishi et al. (2007) verified that participants practicing long-term meditation demonstrated greater skills in regulating their executive control attention compared to those in a control group. Teper et al. (2013) found that participants who scored high on the acceptance facet of mindfulness committed fewer errors on a Stroop task, a canonical measure of executive control. The current study extended previous research and suggested that long-term tai chi experience could improve elders’ executive control abilities by reducing functional connectivity within the executive control network, which might be related to the non-evaluative stance and acceptance toward inner experiences of tai chi practitioners.

Furthermore, the present study found a relationship between functional connectivity within the executive control network and sensitivity to objective outcomes during the sequential risk-taking task for long-term tai chi practitioners. Specifically, reduced functional connectivity between the DLPFC and the MFG was associated with less sensitivity to objective outcomes, that is, stronger ability of emotion regulation, or strategies that could influence which emotions arise and how these emotions were experienced and expressed (Gross and Thompson, 2007). Neuroimaging studies indicated that both the DLPFC and the MFG were involved in the top-down control of emotion regulation (MacDonald et al., 2000; Liu Z. et al., 2017). Notably, the current study also revealed that the functional connectivity between the DLPFC and the MFG in the tai chi group fully mediated the impact of non-judgment of inner experience on their sensitivity to outcomes. Previous studies failed to measure the relationship among meditation, functional connectivity within the executive control network, and emotion-regulation ability. To the best of our knowledge, this was the first imaging study showing that for long-term tai chi practitioners, the modulation of non-judgment of inner experience on their emotion regulation was achieved through decreased functional connectivity between the DLPFC and the MFG.

## Limitations

This study did not mention the exercise intensity of the control or tai chi groups. As tai chi combines meditation with physical exercise of light-to-moderate intensity, it is possible that the exercise intensity in the control group was much higher than in the tai chi group even if the exercise time per day was similar between these two groups. The difference in exercise intensity between the tai chi and control groups may have contaminated the research results. This study’s argument will be strengthened by clarifying if the intensity and modality of the exercises practiced by the controls were similar to the physical components of tai chi.

## Conclusion

The current resting-state fMRI study aimed to examine whether the impact of the meditative component of tai chi on emotion regulation was mediated by functional connectivity within the executive control network. Behaviorally, for older adults, long-term tai chi experience can improve both non-judgment of inner experience and emotion-regulation ability. Moreover, the non-judgment of inner experience of the tai chi practitioners was positively correlated with their emotion-regulation ability. On the neural level, long-term tai chi experience could reduce the functional connectivity between the DLPFC and the MFG, which was a reflection of stronger executive control function in elders. Interestingly, in older adults with long-term tai chi experience, the functional connectivity between the DLPFC and the MFG fully mediated the impact of non-judgment of inner experience on their emotion-regulation ability. The current study showed that the modulation of non-judgment of inner experience on long-term tai chi practitioners’ emotion regulation was achieved through decreased functional connectivity within the executive control network.

## Author Contributions

XG, ZL, and LL designed the experiments, assisted with the interpretation of the data, and wrote the manuscript. ZL and YW programmed the experimental scenario, conducted the experiments, and analyzed the data. All of the authors read and approved the final version of the manuscript for submission.

## Conflict of Interest Statement

The authors declare that the research was conducted in the absence of any commercial or financial relationships that could be construed as a potential conflict of interest.

## Footnotes

1 http://rfmri.org/DPARSF

2 http://www.fil.ion.ucl.ac.uk/spm

3 http://www.restfmri.net
